# Shaping the Future of Research: a perspective from junior scientists

**DOI:** 10.12688/f1000research.5878.2

**Published:** 2015-01-09

**Authors:** Gary S. McDowell, Kearney T. W. Gunsalus, Drew C. MacKellar, Sarah A. Mazzilli, Vaibhav P. Pai, Patricia R. Goodwin, Erica M. Walsh, Avi Robinson-Mosher, Thomas A. Bowman, James Kraemer, Marcella L. Erb, Eldi Schoenfeld, Leila Shokri, Jonathan D. Jackson, Ayesha Islam, Matthew D. Mattozzi, Kristin A. Krukenberg, Jessica K. Polka

**Affiliations:** 1 Department of Biology, Center for Regenerative and Developmental Biology, Tufts University, Medford, MA, 02155, USA; 2 Department of Molecular Biology and Microbiology, Tufts University, Boston, MA, 02111, USA; 3 Department of Systems Biology, Harvard Medical School, Boston, MA, 02115, USA; 4 Department of Computational Biomedicine, Boston University School of Medicine, Boston, MA, 02118, USA; 5 Department of Biology, Brandeis University, Waltham, MA, 02453, USA; 6 Department of Pathology, Brigham and Women's Hospital and Harvard Medical School, Boston, MA, 02115, USA; 7 Wyss Institute for Biologically Inspired Engineering, Harvard Medical School, Boston, MA, 02115, USA; 8 Human Nutrition Research Center on Aging, Tufts University, Boston, MA, 02111, USA; 9 Department of Biology and Howard Hughes Medical Institute, Massachusetts Institute of Technology, Cambridge, MA, 02139, USA; 10 Department of Molecular and Cellular Biology, Harvard University, Cambridge, MA, 02138, USA; 11 Synthetic Biology Center, Department of Biological Engineering, Massachusetts Institute of Technology, Cambridge, MA, 02139, USA; 12 Division of Genetics, Department of Medicine, Brigham and Women’s Hospital and Harvard Medical School, Boston, MA, 02115, USA; 13 Department of Neuroscience, Brandeis University, Waltham, MA, 02453, USA; 14 Department of Obstetrics and Gynecology, Boston University School of Medicine, Boston, MA, 02118, USA

**Keywords:** biomedical research, funding, training, publishing

## Abstract

The landscape of scientific research and funding is in flux as a result of tight budgets, evolving models of both publishing and evaluation, and questions about training and workforce stability. As future leaders, junior scientists are uniquely poised to shape the culture and practice of science in response to these challenges. A group of postdocs in the Boston area who are invested in improving the scientific endeavor, planned a symposium held on October 2 ^nd^ and 3 ^rd^, 2014, as a way to join the discussion about the future of US biomedical research. Here we present a report of the proceedings of participant-driven workshops and the organizers’ synthesis of the outcomes.

## Executive summary

The Future of Research Symposium, held in Boston in October 2014, was born out of a desire on the part of junior scientists to influence discussions about the future of biomedical research in the United States. We the organizers believe that current trainees in academic research represent a talented pool of people contributing to scientific progress. This pool, however, is far larger than the current academic system is able to support in the long term. As structural forces governing the funding and administration of science push many graduate students and postdocs out of research, the public funds supporting their training are poorly repaid.

While scientists continue to advocate for increased funding, they must also create a scientific enterprise that is sustainable with the current resources. A sustainable long-term investment in science, including the young people who carry it out, is essential to the long-term economic and social interests of the US. In the experience of the organizers, the current hyper-competitive environment stunts scientific curiosity and productivity, breeds fabrication and carelessness in the publication of data, and leads to a waste of valuable resources and intellectual capital. In all of our discussions of these problems, we have kept two goals in mind: to maximize the potential for wide-ranging and fundamental scientific discovery; and to minimize the loss of talented young researchers who can contribute greatly to science.

In addition to voicing our concerns, we junior scientists recognize that we need to become more aware of the issues facing the research enterprise, comprised of academia, industry, publishing, and government. To accomplish this, the initial sessions of the symposium consisted of a series of talks and panel discussions from leaders who have been outspoken about the challenges that science faces. These were followed by workshops designed to elicit the opinions and ideas of participants, largely postdocs and graduate students, on problems and solutions surrounding training, the structure of the research workforce, funding, and incentives and rewards in science. We present the outcomes of those discussions in this report, conveying in aggregate many young biomedical scientists’ concerns about the sustainability of the research enterprise and our hopes for change.

From the many ideas presented in the workshops and continued discussions among the organizers, we have distilled the following three principles to guide future activities towards scientific reform:

1. We recommend increased **connectivity** among junior scientists and other stakeholders to promote discussions on reforming the structure of the scientific enterprise.
2. We advocate for increased **transparency**. This includes the number and career outcomes of trainees, as well as the expectations of the balance between employment and training in individual postdoctoral appointments.
3. We call for an increased **investment** in junior scientists, with increased numbers of grants that provide financial independence from Principal Investigator (PI) research grants, and increased accountability for the quality of training as a requirement of funding approval.

As the engine of academic research, junior scientists must claim a voice fitting their role as major stakeholders in the scientific enterprise. Equally, junior scientists must be educated about their role so that they have the context necessary to make a well-informed contribution and to effectively advocate for their interests. By bringing our concerns into the conversation that guides policy, the dialogue will be enriched with diversity and fresh perspectives. We encourage our peers to continue this conversation, engage their colleagues, and to get involved in shaping the Future of Research.

## Context for the Future of Research Symposium

*““The government should provide a reasonable number of undergraduate scholarships and graduate fellowships in order to develop scientific talent in American youth. The plan should be designed to attract into science only that proportion of the youthful talent appropriate to the needs of science in relation to the other needs of the nation’s high priority”. **And I think that is one of the places where we have in biomedical science gone astray**”.*
Shirley Tilghman, quoting Vannevar Bush, at a meeting of the President’s Council of Advisors on Science and Technology (PCAST), September 19 2014, (“ PCAST Meeting 2014”, 2014).

A large portion of the nation’s science and engineering research is carried out by graduate students and postdocs. Because of this, the current culture of training places a heavy emphasis on research and publications, at the expense of “soft skill acquisition” or career development.

In the US, pre-doctoral training in the biomedical sciences takes 6.5 years on average ( Figure 3 of ( Biomedical Research Workforce Working Group, 2012)), and includes research experience culminating in a PhD dissertation. This process is overseen by a committee of 3–5 faculty members and requires the development of some core skills.

In contrast, it is notoriously difficult to determine how many postdoctoral scholars there are, let alone what kind of training they are or should be receiving. The National Institutes of Health (NIH) and the National Science Foundation (NSF) define a postdoctoral scholar as “an individual who has received a doctoral degree (or equivalent) and is engaged in a temporary and defined period of mentored advanced training to enhance the professional skills and research independence needed to pursue his or her chosen career path” ( Bravo & Olsen, 2007). Most postdoctoral “trainees” conduct research under the supervision of a single Principal Investigator (PI), and there are no explicit guidelines to determine what training a postdoc should receive or when this training is complete. In reality, postdoctoral research is often not a training period at all, but a time when experienced junior researchers contribute significantly to the goals of a PI’s grant. There is no expectation of specific training, and no defined period in which the training takes place: “training” ends only when the postdoc takes another job.

In spite of the number of years spent in pre- and postdoctoral training, the organizers perceive that many scientists feel that they are inadequately prepared for any job other than conducting research. Many feel they are unaware of what jobs they should be training for, let alone what skills those jobs require. One common complaint we hear among our colleagues is that scientists are not being prepared for non-faculty positions, yet in the organizer’s experience many new faculty appear unprepared for their non-research responsibilities (such as managing employees and budgets or teaching and we feel that we are not even being properly trained to become future faculty.

### Where did all the graduate students and postdocs come from?

While the number of US graduate students in biomedical science have increased from about 46,500 in 1993 ( Table B-18 in ( National Science Foundation, 1994)) to almost 71,000 in 2012 ( Table 16 in ( National Science Foundation, 2014)), the fraction of PhDs in life sciences in a tenure-track position 5 years post-PhD decreased from 17.3% (1993) to 10.6% (2010) ( Table 3–18 in ( National Science Board, 2014)). There has also been a tremendous shift in the job market outside of academia over the past decades, with a general slowdown and even contractions in government and industry. This situation has long been deemed unsustainable by many senior academics ( Bourne, 2013a; Stephan, 2012a; Stephan, 2012b; Teitelbaum, 2008).

With the number of graduate students increasing faster than the number of faculty positions ( Figure 1 in ( Schillebeeckx *et al.*, 2013)), it is unsurprising that the NIH estimates that the number of postdoctoral researchers also doubled during that time. However, estimates of the number of postdocs vary drastically. The National Research Council puts the number of postdocs at just over 50,000 ( National Research Council (US) Committee to Study the National Needs for Biomedical, Behavioral, and Clinical Research Personnel, 2011), but the NIH states that this could be under-estimated by as much as a factor of two ( Biomedical Research Workforce Working Group, 2012). According to a recent report by the National Postdoctoral Association (NPA), the NPA’s 167 member institutions alone estimate that their postdoc offices serve about 79,000 postdocs ( Ferguson *et al.*, 2014).

### Where do graduate students and postdocs actually go?

Data from the NSF Survey of Doctorate Recipients suggests that the US-trained biomedical PhDs “who do the longest postdocs are the ones who go on to tenure-track academic research careers” ( Rockey, 2012). However, in spite of the number of scientists remaining in long postdocs in the hopes of landing a tenure-track faculty position, the data show clearly that academia is an “alternative” career, not the default. In 2010, less than 15% of US-trained science, engineering and health sciences postdocs had obtained a tenure-track faculty position within 5–7 years of completing their PhD ( Sauermann & Roach, 2012). The rest of the job market encompasses many fields that are expanding and that we the organizers believe can benefit from the trained minds of PhDs and postdocs. These include (but are not limited to): consulting for life sciences, biotech and biopharmaceutical industries, sales and marketing of technologically advanced products, regulatory affairs, science policy, science communications, and intellectual property.

Even though the majority of postdocs will do something other than become tenure-track faculty members, the default assumption of many PIs (and their mentees) remains that graduate students and postdocs will follow their mentors’ career trajectory and acquire an academic faculty position at a research-intensive institution ( Sauermann & Roach, 2012). The data show that by the end of their PhD training, only 50% of graduate students want to become academics, and that expectations change over time: a faculty position becomes less attractive over the course of a PhD, in spite of active encouragement by advisors ( Sauermann & Roach, 2012).

Thus, many junior scientists want, and most will obtain, non-faculty jobs. However, we the organizers feel that few young scientists and their faculty mentors know what careers are actually available, let alone what skills those jobs require or how to obtain them. The mismatch between scientists’ career expectations and the realities of the job market has led to extended occupancy of postdoc positions ( Biomedical Research Workforce Working Group, 2012) and we believe this leads to highly inflated expectations from academic employers for prior productivity.

### How does the funding system contribute to workforce and training problems?

In the US, the funding system has had a profound impact on the structure of universities and academic and applied research departments, and how the time of principal investigators and young scientists is spent.

As early as 2003, the rapid increase in funds over the previous decade was generating questions about where trainees would end up in the absence of a concomitant increase in academic positions ( Russo, 2003). In response to these concerns, there have been calls for institutions to become more responsible for funding “hard-money” faculty positions, and to increase NIH incentives for doing so, rather than relying on external sources of funding for “soft-money” positions ( Alberts, 2010). These problems were left unresolved, however, and now that there has been a contraction in funding they have become immediate. For institutions and individual researchers attempting to make long-term decisions, financial uncertainty makes planning very challenging. It is clear that simply putting more money into the system would provide only a temporary fix, not a long-term solution to the systemic problems with academic research ( Alberts *et al.*, 2014; Martinson, 2007). Among these problems is an implication (expressed through the growth of, and reliance on, graduate student and postdoc populations) that the enterprise will grow exponentially. In the face of stagnant funding, this growth has instead intensified competition for jobs, grants, and publications ( Alberts *et al.*, 2014).

### What’s wrong with competition?

An assumption of many industries is that increased competition between groups or individuals yields largely beneficial results. However, academic science in the US was essentially founded on Vannevar Bush’s principle of the “supreme importance of affording the prepared mind complete freedom for the exercise of initiative” ( Bush, 1945). These two principles are incompatible.

Indeed, we organizers believe that the problems caused by the current unsustainable workforce are threatening the very foundations of scientific research. The high stakes and low expectations of success prevalent throughout biomedical research, from grant applications to hiring decisions, promote academic dishonesty ( Lang, 2013). Also, success in grant applications and career progression relies heavily on publications ( van Dijk *et al.*, 2014). This can lead to hyper-competition for “high-impact” publications and in some recent cases, a lack of truth in publishing ( Nosek *et al.*, 2012; Sovacool, 2008). Competition also encourages scientists to present data in the most optimistic light, and to include only data that lead to a clean and understandable conclusion. As postdocs, we see and experience these pressures first-hand. The pressure to publish needs to be balanced with incentives for rigorous and honest scientific communication.

However, dishonesty is not the only problem threatening the integrity of academic literature. Part of the scientific endeavor is to provide checks and balances, reproduce results, and highlight when reproducibility fails. However, it is difficult (and unrewarding) to publish the results of replicative experiments or negative data, and there is a worrying trend in the lack of reproducibility in some forms of analysis; this issue was recently highlighted with regard to the widely-used technique of fluorescence-activated cell sorting ( Hines *et al.*, 2014). Some journals have made a call specifically for papers reporting negative data, and there are indications that the NIH may be looking to drive more studies testing whether data can be reproduced ( Collins & Tabak, 2014).

Hyper-competition can also discourage creative thinking and risk-taking, strong foundations of the scientific endeavor ( Alberts *et al.*, 2014). Rather than grant applications for innovative, breakthrough science, we have observed that hyper-competition results in “safe” applications, driving incremental, slow improvements on existing knowledge ( Alberts *et al.*, 2014). It blunts the blade of science, preventing it from piercing through existing ideas and paradigms to expose new frontiers.

## Junior scientists must join the debate

A range of problems with the biomedical research system in particular have been the subject of increasing alarm in the scientific community ( Alberts *et al.*, 2014; Bourne, 2013a; Bourne, 2013b; Bourne, 2013c). While the focus has mostly been on US academic science, many of the problems are universal. These issues are not just relevant to those inside academia: due to their importance to national competitiveness, they are increasingly featured in the popular media as well ( Harris, 2014a; Harris, 2014b; Harris, 2014c; Harris, 2014d).

The public debate surrounding these issues has so far been led by senior members of academia ( Alberts *et al.*, 2014). One group that has yet to contribute significantly to the discussion is the largest group of researchers affected: graduate students and postdocs. Boston-area postdocs organized the Future of Research Symposium to raise awareness of the difficulties faced by young scientists and to provide a venue for further discussion and problem-solving during a set of interactive workshops.

We issued a call-to-arms to our peers to announce what we were doing, and to emphasize our view that young researchers should have a say in shaping the future direction of the research endeavor ( McDowell *et al.*, 2014a). To achieve our goal of giving a voice to the aspirations of young researchers, we synthesized the current issues that have been identified as obstructing the progress of scientific research into four focus areas: funding for biomedical research, training of the scientific workforce, the structure of the workforce, and incentives and rewards for scientists ( McDowell *et al.*, 2014c). Interactive problem-solving workshops honed in on each topic to explore the problems and propose solutions with the aim of formulating a response that we can provide to the larger scientific community. This document is the first to begin disseminating that response to foster and foment further discussion and action. Here we present the problems identified and tentative solutions suggested by participants in the workshops. We then discuss areas identified through ongoing discussions as requiring the most urgent action from young scientists to improve the Future of Research.

*“To be creative…emphasize new possibilities by disclosing those hidden episodes of the past when, even if in brief flashes, people showed their ability to resist, to join together, occasionally to win”*.
Howard Zinn ( Zinn, 2014)

## Symposium organization

The Future of Research Symposium was organized by a group of postdoctoral scholars from universities in the Boston area, including Boston University, Harvard University, Harvard Medical School, Tufts University, Brigham and Women’s Hospital, the Massachusetts Institute of Technology, Brandeis University, and the Dana Farber Cancer Institute. The symposium was hosted at Boston University through a partnership with Boston University’s Graduate Women in Science and Engineering (GWISE).

Speakers from academia and industry who have led national discussions participated. Henry Bourne opened the symposium with a keynote outlining the changes he thinks must be made to the scientific infrastructure. A panel comprising Sibby Anderson-Thompkins (Director, Office of Postdoctoral Affairs, University of North Carolina at Chapel Hill), Galit Lahav (Associate Professor, Harvard Medical School), Graham Walker (American Cancer Society Professor, HHMI Professor, Massachusetts Institute of Technology), David Glass (Executive Director, Novartis Institutes for Biomedical Research), and Richard Roberts (Chief Scientific Officer, New England Biolabs) summarized weaknesses and potential improvements in the current training system. A second panel comprising Marc Kirschner (John Franklin Enders University Professor of Systems Biology, Harvard Medical School), Michael Teitelbaum (Senior Research Associate, Harvard Law School), Naomi Rosenberg (Dean of the Sackler School of Graduate Biomedical Sciences, Tufts University), and Cynthia Furhmann (Dean of Career & Professional Development in the Graduate School of Biomedical Sciences, University of Massachusetts Medical School) discussed issues pertaining to the scientific workforce and their implications for the future of science in the United States.

While we did not strictly monitor the attendance at the symposium, registration data suggested that the majority of participants were postdocs and graduate students. Of 658 registrants, 344 were postdocs, 140 were graduate students, and the remainder included a mix of professors, instructors, journalists, administrators, research technicians, and research scientists from both academia and industry.

For detailed information on the requirements for preparing a symposium please see: *The Logistics of Organizing the Future of Research Symposium* ( Mazzilli *et al.*, 2014).

## Participant-led workshops at the Future of Research Symposium

In order to focus the aims of the workshops, participants were invited to complete an anonymous survey of their ideas about how science should be conducted and supported, and the problems they identified with the current system. The results of this survey can be found in Appendices 1A & 1B in Dataset 1.

We considered the results of the survey as indicative of a general dissatisfaction with the current research paradigm, but not necessarily prescriptive of specific and comprehensive solutions. The output of this survey is informative in gauging the general opinion of educated, disciplined, and curious people pursuing science in the US.

Symposium workshops were designed to allow participants to discuss issues identified as obstructing the progress of scientific research as well as to provide opportunities to discuss potential solutions.

Each workshop was overseen by three to four moderators from the organizing committee who provided some background on the current system and posed the specific objective for each session. The four objectives were to ask:

- How can trainees be better prepared for careers in science in 2014?
- How should the supply of postdocs and graduate students be matched to the demand for jobs in order to create a sustainable workforce?
- How can the funding of academic research be structured to promote desired outcomes such as the discovery of basic knowledge, finding applications of knowledge for the betterment of society, and training the next generation of scientists?
- How can the current system of incentives be fixed so that scientists and institutions are rewarded for the behaviors that are believed to support good science?

Workshops were broken down into two separate 90-minute sessions. The number of participants per topic per session was typically between 20 and 30. Individual participants were asked to write down the perceived problems with the current system on post-it notes and to post them on the wall. Working as a group, participants categorized these individual responses and identified major themes. Participants were then asked to individually write down possible solutions to the identified problems. This was once again done on post-it notes. Solutions were categorized according to the level of implementation, ranging from actions that can be accomplished by individual graduate students and postdocs to those requiring action from society as a whole. If time permitted, participants voted on solutions they found most compelling and discussed the pros and cons of these solutions further. Generally, there was not sufficient time to discuss any potential solutions in depth. We view these sessions primarily as a way to begin debate, not to end it.

**Chart 1.**
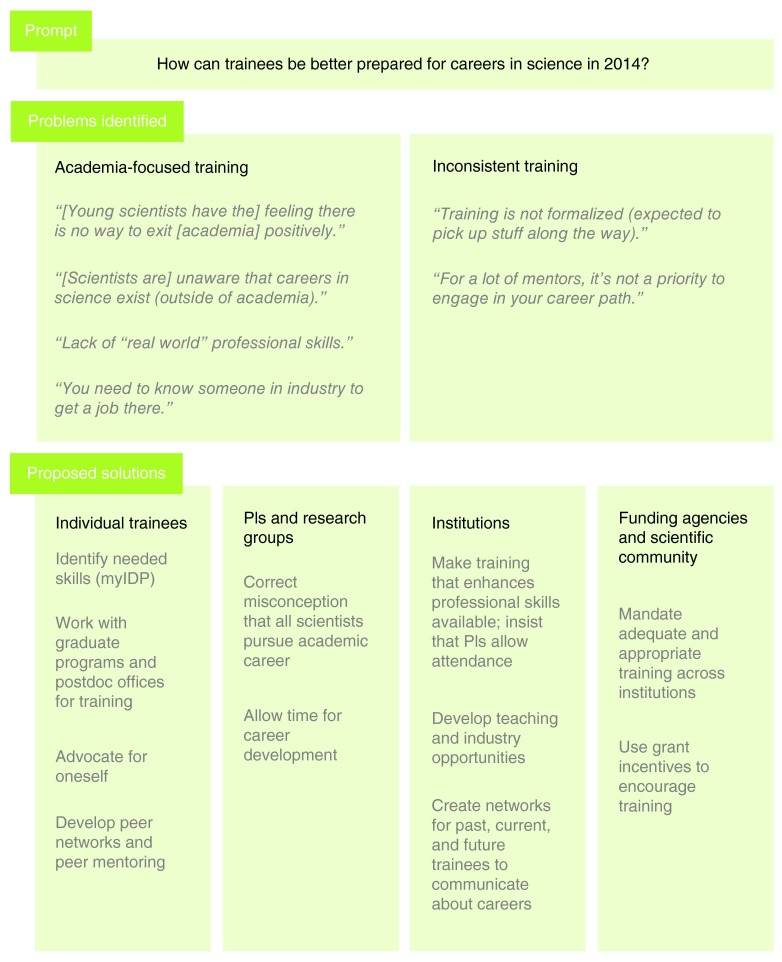
Summary of the outcomes of the training workshop.

The workshops identified a large number of problems and potential solutions, many of which were raised repeatedly, though the immediate topic of conversation varied. In the following sections, we summarize the identified problems and proposed solutions in Chart 1– Chart 4. We also list the identified problems and proposed solutions in more detail, without necessarily endorsing each possible solution, together with a few common themes distilled from each workshop. The raw data for each workshop can be found in Appendices 2A–D.

At the end of each workshop, participants were asked to fill out a short exit survey (full text in Appendix 3; individual comments from each workshop in Appendices 3A–D in Dataset 1). The survey was designed to address three objectives; 1) to assess how well the workshop format was working and how it could be improved; 2) to determine whether or not participants felt they had reached a consensus during the workshop, and to gauge the importance participants placed on reaching consensus about these issues; and 3) to solicit specific suggestions they might have about next steps to be taken after the symposium. The results of the survey are summarized in Appendix 3 in Dataset 1.

## Training for careers in science in 2014

The outcome of this workshop highlighted that the current culture of training places heavy emphasis on research and publications, leaving little time for “soft skill” or career development. Postdoctoral “training” is a misnomer: as one participant put it, “ *If you’re going to call me a trainee, then train me”*. Rather than force everyone to be trained for the same (academic) career path, institutions should provide opportunities for trainees to acquire skills that are useful in multiple career paths, and PIs should be required to allow trainees access to these training opportunities.

Postdocs were consistently called “the lost people” and “the invisible people”. Postdocs do not yet have a coherent voice, and we must change this. Postdoctoral associations should be advocating for access to training, both in provision and time allowance, in their institutions. The National Postdoctoral Association should have a stronger voice in advocating for postdoctoral training at a national level. Trainees should involve themselves with their learned societies to influence policy. Finally, researchers should be involving the wider public: to describe what can be given to society, to demonstrate their value, and also to highlight the waste of human capital and taxpayer money that goes into funding inadequate training ( Chart 1).

## Problems identified

Participants identified problems with the current training system in the following key areas (Appendix 2A in Dataset 1):

**Culture of academia-focused training:** The prevailing view of training focuses heavily on academia, where few scientists can obtain positions. This creates a sense of failure for those leaving academia.

*“[Young scientists have the] feeling there is no way to exit [academia] positively”*.

**Absence of awareness of non-academic job opportunities:** Scientists have limited knowledge of careers outside of academia that require scientific training. They are not aware of the kinds of jobs they may be qualified for; the skills these different jobs may require; and how to successfully apply for these jobs.

*“[Scientists are] unaware that careers in science exist (outside of academia)”*.

**PIs are not equipped to advance their mentees’ careers:** PIs have little incentive to act as a mentor for a trainee’s career development, and limited training that would make them competent to do so.

*“For a lot of mentors, it’s not a priority to engage in your career path”*.

**Informal training leads to inconsistent training:** There is a lack of standardized training for any scientific career, be it academic or non-academic. PIs require multiple skills learned only from experience; current training was described as “spotty” and “overly specialized”. Training standards are highly variable between institutions and research groups.

*“Training is not formalized (expected to pick up stuff along the way)”*.

**Lack of professional skills training:** Current training fails to teach skills that can be applied to both academic and non-academic careers, including people management, networking, writing, and presentation skills. Scientists learn to conduct research, but not to manage a research group.

*“Lack of “real world” professional skills”*.

**Little or no training on transitioning to industry:** There is a dearth of training about how to transition from academia to industry. There are too few internship programs providing experience in industry.

*“You need to know someone in industry to get a job there”*.

## Proposed solutions

**Individual graduate students and postdocs**

- Graduate students and postdocs can identify the skills they need to develop (such as via the my Individual Development Plan (myIDP) tool ( Fuhrmann *et al.*, n.d.)), then collaborate with each other and with graduate programs and postdoctoral offices to acquire training.
- Postdocs should advocate for themselves, network with each other, and provide mentorship to each other.

**PIs and research groups**

- We must correct the misconception that all scientists will pursue an academic career.
- PIs should allow time for career development; recent data suggests this will not detract from research productivity ( Rybarczyk *et al.*, 2011; Strategic Evaluations, Inc., 2014).

**Institutions**

- Institutions should make adequate, appropriate training available and insist that PIs allow attendance. “Adequate, appropriate training” should enhance the professional skills that graduate students and postdocs have identified as important for their chosen careers.
- Institutions should develop teaching and industry opportunities.
- Institutions could create networks that allow for past, current and future trainees to communicate about careers.

**Funding agencies and the scientific community**

- Availability of adequate, appropriate training should be mandated across all institutions.
- Grant incentives should be used to encourage PIs to facilitate adequate training.

## Towards a sustainable workforce

**Chart 2.**
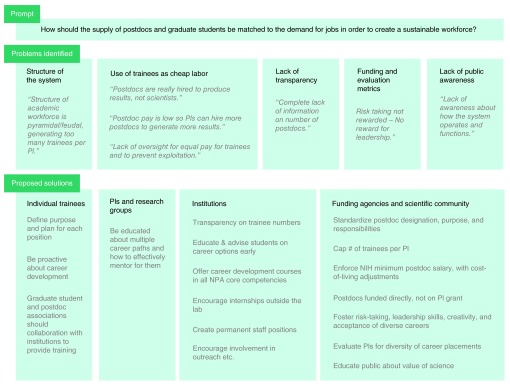
Summary of the outcomes of the workforce workshop.

There is a clear imbalance between the number of young scientists and the number of jobs available in research. This schism has been widening for the past few decades and producing stress on the scientific workforce which, if unaddressed, will result in a decline in the number of productive young scientists. The fundamental structural flaws in the system need to be addressed; otherwise, as we have seen in the past, simply increasing funding will only postpone and worsen the problem.

Young scientists need to be engaged in the debate about these changes and advocate for them. They need to come together in collaboration with institutions and the federal government to enforce and implement these changes with a clear discussion of all possible outcomes of these changes.

Ultimately the scientific enterprise will grow if the workforce supply and demand are balanced in a sustainable and dynamic fashion, with complete transparency. We can build a highly efficient and productive scientific enterprise if scientists, institutions, governments and industry are all involved and invested in making the necessary changes to the workforce ( Chart 2).

## Problems identified

Participants identified problems with the structure of the workforce in the following key areas (Appendix 2B in Dataset 1):

**Structure of the system:** PIs currently train junior scientists (multiple trainees per PI) in their own image, that is, for a career in academia, though only a small minority will obtain tenure-track faculty positions. Most PIs know little about non-academic careers, even though these comprise the majority of future careers for today’s postdocs. These non-faculty careers are often still looked down upon by those in academia. There is little attention given to training for the careers that the majority of junior scientists will eventually pursue.

*“Structure of academic workforce is pyramidal/feudal, generating too many trainees per PI”*.

**Use of graduate students and postdocs as cheap labor:** Junior scientists are primarily treated as cheap labor rather than as participants in a well-rounded training program that prepares participants for a range of clearly identified career options. Postdocs are conflictingly defined as trainees and employees in different situations, which is made possible by the lack of a standardized designation for postdocs and of a clear definition of their duties and responsibilities. There is also no oversight over the number of graduate students and postdocs and whether that number is appropriate given the perceived job market demand. Additionally, there was consensus that funding postdocs through research grants puts them in a vulnerable position and encourages low postdoc salaries allowing for the use of funds elsewhere.

*“Postdocs are really hired to produce results, not scientists”*.
*“Postdoc pay is low so PIs can hire more postdocs to generate more results”*.
*“Lack of oversight for equal pay for trainees and to prevent exploitation”*.

**Lack of transparency:** Problems with workforce sustainability are perpetuated by a lack of information and awareness about the situation, particularly amongst incoming graduate students who seek the increasingly rare academic careers that are still treated as the default career choice by many graduate programs.

*“Complete lack of information on number of postdocs”*.

**Funding and evaluation metrics:** Current metrics of evaluation, which are based on the number and impact factor of publications, have resulted in a culture of hyper-competitiveness which discourages creativity, co-operation, risk-taking and original thinking.

*“Risk taking not rewarded – No reward for leadership”*.

**Lack of public awareness:** Participants also felt a pressing need to make the general public aware of what a scientist really is and what she does, and to more effectively communicate the value of science to the US economy and to humanity as a whole.

*“Lack of awareness about how the system operates and functions”*

## Proposed solutions

**Individual graduate students and postdocs**

- Each postdoctoral position should have a defined purpose, including a plan for enhancing the professional skills required in that postdoc’s chosen career path.
- Graduate students and postdocs should be proactive about getting career information and carrying out self-evaluation, and discussing these with their mentors. They could also assemble their own career development committee, made up of mentors from various careers of interest.
- Graduate student and postdoc associations should collaborate within and between institutions to provide career information and training.

**PIs and research groups**

- PIs should be educated about career paths and trends in the biomedical workforce and how to effectively mentor students and postdocs for available jobs.

**Institutions**

- Institutions should be transparent about the number and funding source of graduate students and postdocs.
- Admission of graduate students could take into consideration their career path and the objective of their training.
- Incoming graduate students should be educated about career options and provided with career development advisors.
- Institutions should offer career development courses in all areas of the National Postdoctoral Association core competencies ( The National Postdoctoral Association Core Competencies Committee, n.d.).
- Permanent staff scientist positions should be created with funding structures that remove the competition between the staff scientist and cheaper postdocs or graduate students.
- Scientists’ involvement in outreach, politics, and entrepreneurship should be encouraged.

**Funding agencies and the scientific community**

- There should be a standardized designation for all postdocs, irrespective of funding source.
- The purpose and responsibilities of postdocs should be clearly defined.
- Caps should be placed on the number of junior scientists per PI.
- All postdocs should receive at least the NIH minimum salary, with a geographical cost-of-living adjustment ( US Office of Personnel Management, n.d.), and certain basic benefits.
- Funding for postdocs should not be tied to PI research grants.
- The hyper-competitive publish-in-high-impact-journals-or-perish culture should be discouraged and risk-taking, leadership skills and creativity fostered instead.
- As a community, scientists should campaign to educate the public about who scientists are, what they do, and the value of their work.
- Within the academic scientific community, we should foster acceptance of non-academic career path choices.
- PIs should be positively evaluated for diversity of successful career paths taken by their trainees, and not just on the number of trainees that they have placed in research-track careers.

## Funding innovation and training

**Chart 3.**
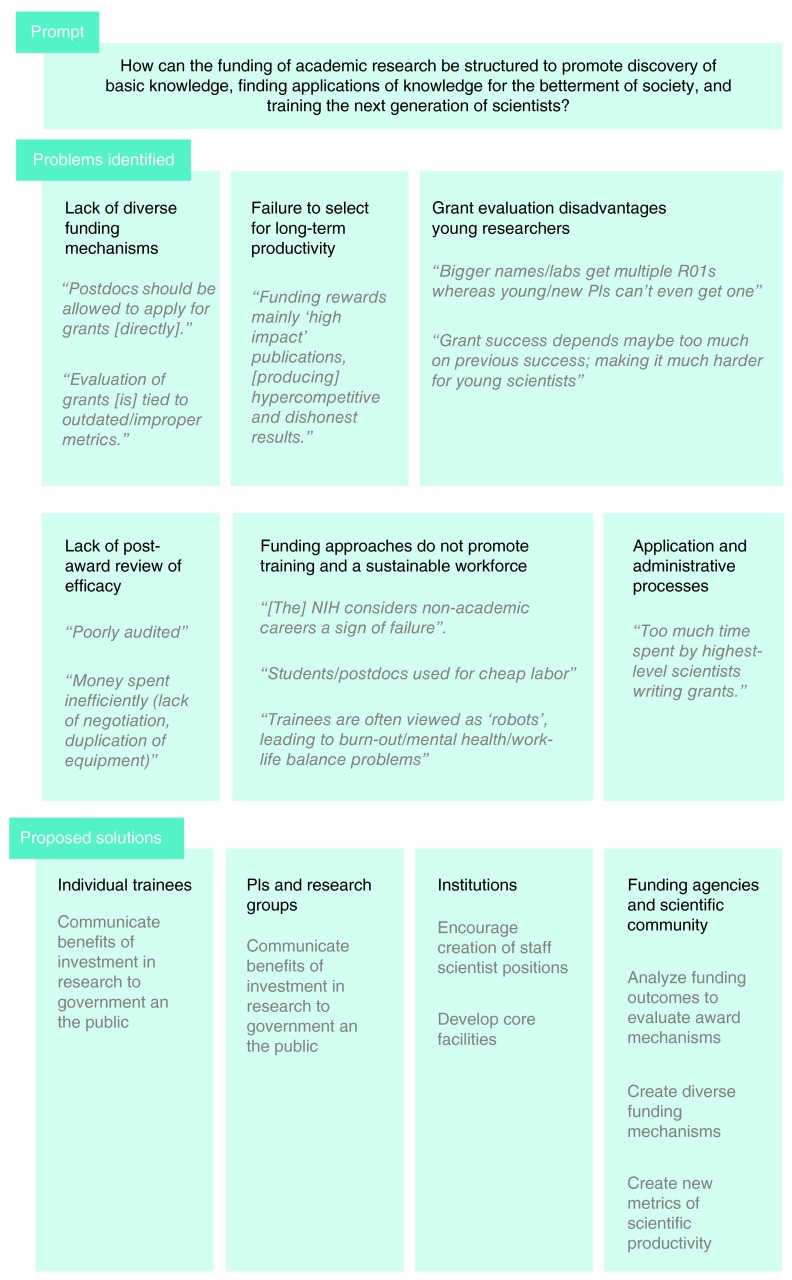
Summary of the outcomes of the funding workshop.

Overall, we would characterize the output of this workshop as a call by young researchers for an increase in the efficiency and reproducibility of science by developing new measures of the quality of research output and of individual researchers’ productivity, and incorporating these criteria into the approval of grants. Participants seemed to agree that this approach, along with some of the other recommendations indicated, would more adequately reflect the priorities of federally-funded science and encourage young researchers to continue careers in basic research ( Chart 3).

## Problems identified

Participants identified problems with funding in the following key areas (Appendix 2C in Dataset 1):

**Funding mechanisms were considered insufficiently diverse:** Many participants were in favor of extending the time scales of awarded grants, and cited a need for alternative mechanisms to workhorse grants like the R01, that might permit research projects with alternative aims and organization. In addition, the NIH grant review cycle was seen as inefficiently slow and too bureaucratic to effectively support innovative work. Participants were frustrated at the way that funding agencies were considered to encourage incremental steps in research, thereby discouraging paradigm shifts. They also expressed concern that current funding mechanisms “kill novel ideas by emphasizing preliminary results”.

*“Postdocs should be allowed to apply for grants [directly]”*
*“Evaluation of grants [is] tied to outdated/improper metrics”*

**Funding priorities fail to select for long-term productivity:** Congressional and institutional trends heavily influence how research money is distributed, such that too much of the available funding is oriented towards ephemerally popular topics, while mature, yet important, research fields are neglected. Concerns were also raised that recent trends in funding favor applied research at the expense of basic research. These priorities undermine the quality and reproducibility of science that is vital to US interests.

*“Funding rewards mainly ‘high impact’ publications, [producing] hypercompetitive and dishonest results”.*
*“Emphasis on translation and the best ‘new’ idea, not reproducibility”*

**Grant evaluation processes disadvantage young researchers:** Institutional leanings in funding agencies were perceived as resulting in funds that are highly centralized; with large grants being awarded to large, well-established labs.

*“Bigger names/labs get multiple R01s whereas young/new PIs can’t even get one”.*
*“Grant success depends maybe too much on previous success; making it much harder for young scientists”*

**Funding allocation is not subject to post-award review of efficacy:** Participants voiced concerns that the current funding paradigm does not lend itself to quantitative, objective analysis of the productivity or quality of research investments. Name recognition and impact factors were reported as weighing too heavily in single-blind study sections, resulting in funds being allocated unscientifically, with few studies of efficacy or predictors of outcome.

*“Poorly audited”*
*“Money spent inefficiently (lack of negotiation, duplication of equipment)”*

**Approaches to funding were reported as contributing to problems in training and workforce sustainability:** Participants noted an insufficient level of direct funding support for postdocs and graduate students, such as through training grants. They also indicated that, by focusing on research productivity alone, funding mechanisms fail to select for graduate and postgraduate education that would aid trainees in developing the skills that would contribute to success in academia or other environments. Funding agencies were also seen as contributing to the negative way that non-academic careers are viewed.

*“[The] NIH considers non-academic careers a sign of failure”.*
*“Students/postdocs used for cheap labor”*
*“Trainees are often viewed as ‘robots’, leading to burn-out/mental health/work-life balance problems”*

**Grant application and administration processes are problematic:** There was frequent concern regarding the bureaucracy and paperwork involved in applying for and administering grants. Participants characterized the level of effort required to complete auxiliary sections of grant proposals (i.e., outside of specific aims and experimental design) as inefficient, as well as the number of specialized personnel required to submit, review, and administer federal research grants. In addition, several participants found the current peer review system to be insufficiently transparent, and reported that study sections give too little feedback.

*“Too much time spent by highest-level scientists writing grants”.*

## Proposed solutions

**Individual scientists and research groups**

- Scientists should interact more directly with the public and the government to communicate the benefits of investment in research.

**Institutions**

- Staff scientists should be supported by grants in order to improve the continuity and accountability of research results within academic labs.
- Core facilities should be developed to reduce the resources and specialized expertise required in each lab, allowing smaller lab sizes.

**Funding agencies and the scientific community**

- We should analyze basic science funding and outcomes to determine how funding award mechanisms affect science.
- A greater diversity of funding mechanisms serving smaller labs, younger faculty, and even science enthusiasts within the general public, with an emphasis on encouraging shared, collaborative workspace and core facilities, should be developed.
- New metrics evaluating scientific productivity beyond simple impact factor should be established, along with more post-peer-review and scrutiny of results.

## Incentivizing good science

**Chart 4.**
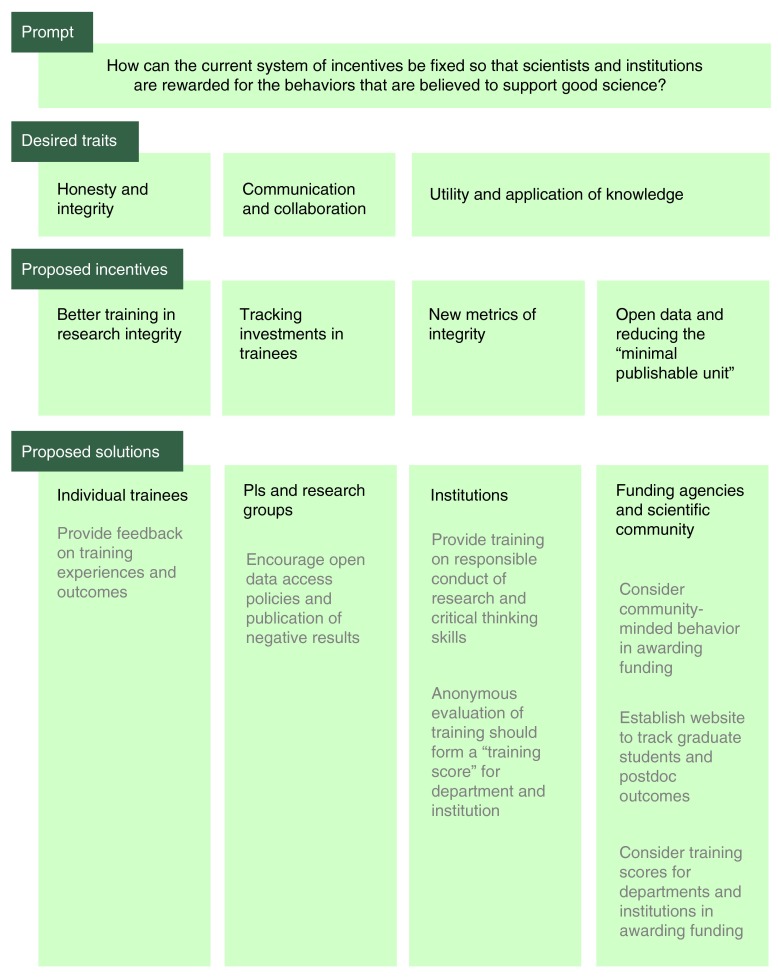
Summary of the outcomes of the incentives workshop.

The output of this workshop was a call by young researchers for incentivization of transparency and honesty in science by developing new metrics and possibly incorporating these criteria into funding mechanisms. In particular, we propose the creation of a website for trainees to anonymously publish feedback on their training experiences and outcomes, ideally using the IDP ( Fuhrmann *et al.*, n.d.) as a framework. Trainees might complete an IDP, then later return to the site to report on their progress. Data, aggregated at the departmental or program level, would form part of a training score for the department and institution. This would permit prospective students and fellows to factor this information into their career decisions, thereby rewarding institutions that place an emphasis on training with improved student and fellow recruitment. Incorporating this score into the grant review process would encourage departments to invest in training. The website could also facilitate publication of institutions’ training plans that outlines available career development opportunities. This could encourage the creation of *de facto* universal standards for training ( Chart 4).

## What we want from scientists and science

Participants identified three major classes of behaviors they wished to see in science (in order of popularity, Appendix 2D in Dataset 1):

**Honesty and integrity:** Scientists should pursue the discovery of truth with honesty and integrity, and to the best of their ability; and should continue pushing the boundaries of human knowledge and asking new questions.

**Communication and collaboration:** Scientists should share information and ideas freely, both among the scientific community and outside of it. Transparency, openness, sharing, the free exchange of ideas and open dialogue among scientists were all identified as key attributes.

**Utility and application of knowledge:** Science should produce useful knowledge that can be applied in beneficial ways, with a responsibility to taxpayers to conduct this research with the greatest efficiency possible.

Participants proposed incentives to encourage the above behaviors:

**Better training in research integrity:** Responsible conduct of research education should begin early in graduate school, and ethics discussions should be commonplace.

**Tracking investments in trainees:** Funding agencies should maintain centralized information on trainee outcomes and make these data available to prospective trainees to encourage investment in students’ and fellows’ education.

**New metrics of integrity:** While current publication metrics encourage flashy publications, metrics should be created to reward integrity and honesty. These measures could include peer review contributions (whether pre- or post-publication); whether qualitative or quantitative, these could influence grant and job applications.

**Open data and reducing the “minimal publishable unit”:** Journals could require data uploads prior to publication and raw data access during revision and/or following publication. This would encourage careful record-keeping and unbiased analysis through the scientific process. Furthermore, many results (especially negative and contradictory results) could be published under new models that do not require the time and resource investment of a traditional paper.

## Proposed solutions

**Individual graduate students and postdocs**

- Graduate students and postdocs should be able to anonymously provide feedback on their training experiences and outcomes, ideally using the IDP as a framework.

**PIs and research groups**

- Open data access policies and publication of negative results should be encouraged.

**Institutions**

- Adequate training on the responsible conduct of research and critical thinking skills should be provided.
- Anonymous evaluation of available training by graduate students and trainees should be aggregated at the departmental level and used to form part of a training score for the department and institution.

**Funding agencies and the scientific community**

- Metrics of community-minded behavior (publishing negative results, peer review activity) should be taken into account when awarding grants.
- A website should be established to track graduate student and postdoc outcomes across institutions.
- A training score for departments and institutions should be considered during grant review.

## Media response and online discussion

The symposium received a wide variety of feedback and responses during and after the event, from both social media and the press, which continues to foster discussion. There has been significant discussion on twitter ( #FORsymp, @FORsymp), in the popular press ( Johnson, 2014)., and in scientific journals (2014). For more on the responses to the Future of Research Symposium see Appendix 4 in Dataset 1.

Update 1. Dataset of Future of Research Symposium Legends describing each file can be found in the text file provided. Two new files have been added (Appendices 1A and 4), one file has been renamed (Appendix 1 has become Appendix 1B) and one file has been modified (Appendix 3).

## Conclusion

The workshops represented an opportunity for junior scientists to come together and discuss problems with the current scientific enterprise, and they produced an abundance of suggested solutions. Given the limited time of the workshops and the varied background of the participants in terms of their perspective on the current system and its challenges, a consensus on specific steps to be taken was not achieved. There were, however, certain common themes that require further discussion; because of the interconnected nature of these issues, effecting change will require a deeper understanding of both the causes of the problems and the effects of the proposed solutions. As a starting point for a larger and longer discussion, we the organizers have distilled three main proposals that can be implemented at all levels, from individual postdocs to institutions such as the NIH.

First, we recommend increased **connectivity** among junior scientists as well as between junior scientists and other segments of the scientific community. Postdocs and graduate students frequently conduct their research in isolation, as their work is rewarded primarily upon the basis of its novelty and independence, and as they are all competitors for a vanishingly small pool of advanced academic positions. The sense of isolation is particularly strong among postdocs, as many uproot themselves from their professional networks to take positions in geographically distant institutions without an accompanying cohort (such as in graduate school). This isolation precludes awareness of larger institutional issues and makes it more difficult for postdocs to advocate for themselves and bring about positive change. Postdocs and graduate students also must connect with other stakeholders in science so as to participate in the ongoing discussions about changes in training, funding, and other important policy issues. Finally, postdocs and graduate students should come together to define their position as major stakeholders in the research enterprise. While as individuals, junior scientists are temporary, replaceable, and largely anonymous, together they constitute the engine of the academic workforce. As such, they need to take collective action to ensure that their interests are protected as they work to maximize scientific output and efficiency ( Cain *et al.*, 2014). Only by bringing all stakeholders together will science be able to effectively grow and adapt to current and future challenges.

Second, we recommend increased **transparency** in trainee numbers and outcomes. Currently, national conversations decrying the “STEM shortage”, as well as a lack of accessible information about the state of the workforce, create skewed perceptions regarding the demand for PhDs among many beginning biomedical graduate students. Students may become aware of the pyramidal structure of the academic workforce only late in their training. To remedy this, the number of graduate students and postdocs at all institutions should be made publicly available, together with information on career outcomes. Collecting and publishing information on career outcomes should be made a condition of an institution receiving NIH funding. Many institutions already collect this information at regular intervals, but lack a centralizing node to distribute it, and to compare the effect of their leadership. These organizations have a moral imperative to share this information; its dissemination will enable informed career and policy decisions. In addition, former students and postdocs should have a forum in which to anonymously report the outcomes of their training and subsequent career moves. Furthermore, there is a significant need to better define the role and purpose of the postdoc position. We advocate for transparency in terms of defining expectations of the balance between employment and training in individual postdoc appointments.

Finally, we call for increased **investment** in postdocs through financial independence from PI research grants and increased accountability for the quality of postdoc training. Currently, many postdocs have little power to freely pursue creative research directions and individual professional development plans, or to negotiate for necessary employment benefits. We propose two possible mechanisms for increasing postdoc autonomy. First, postdocs should not be supported by research grants, but rather exclusively by individual training fellowships. With this increased intellectual independence, postdocs would be allowed to pursue projects of mutual interest to themselves and their mentors. This creates a much-needed line between staff scientists and technicians, who may be paid and directed by research grants, and postdoctoral scholars, who should be focused on training and development. Second, the institutions employing, and the agencies funding, postdocs should seek increased accountability for their training through direct postdoc feedback to the funding agency. These reports of training experience and support given by PIs, departments, and institutions should be used in evaluating grants for award and renewal. Furthermore, some of this information, properly anonymized and aggregated, could be used to create a publicly accessible “training score” for departments; this metric would incentivize excellence in mentoring to maintain competitiveness in recruitment of young, talented scientists.

As the source of future scientific leadership, postdocs and graduate students are uniquely placed to influence the direction and culture of the research enterprise. To be most effective, however, we must educate ourselves about the prevailing conditions affecting the workforce and sustainability of research, and their historical and institutional bases. The voices of junior researchers must command a greater audience in the present discussion; additionally, as we take our places as the next generation of independent academic scientists, we can influence the culture, efficiency, and integrity of research from within. From both the attendance at the symposium and the ongoing coverage of the event and issues discussed, it is clear that junior scientists are invested in and passionate about these issues. We all must now rise to the challenge of taking action to build a sustainable, productive, and equitable scientific community.

## Data availability

F1000Research: Dataset 1. Update 1. Dataset of Future of Research Symposium, 10.5256/f1000research.5878.d41494 ( McDowell *et al.*, 2014d).
