# Supplementary material for: Shaping the Future of Research: a perspective from junior scientists
Source: F1000Res. 2015 Jan 9;3:291. Originally published 2014 Nov 28. [Version 2] doi: 10.12688/f1000research.5878.2 (PMC4304227; doi:10.12688/f1000research.5878.2)
Supplement: Update 1. Dataset of Future of Research Symposium — Legends describing each file can be found in the text file provided. Two new files have been added (Appendices 1A and 4), one file has been renamed (Appendix 1 has become Appendix 1B) and one file has been modified (Appendix 3). [file f1000research-3-6420-s0000.tgz › c07d938a-3bae-4814-adde-c49e101f85f8_Dataset_of_Future_of_Research_Symposium_update_1.zip!/Appendix 3A.odt]

APPENDIX 3A – TRAINING

How could session have been improved?

For white paper, you will need to know: 1) What arguments will work for your intended audience? 2) What will their counterarguments or barriers be? Competing interestes? How do you plan to reach them?
I think it was well-executed, not too much need for improvement.
Come to the session with ideas already so discussion can be on implementation.
More specific topics for alternative careers.
In the end it was mentioned how the proposed solutions are to be implemented. It's important yet no time is left to discuss.
Compile problems/solutions into post-session document to be discussed.
Extremely well-moderated. Great job.
I liked the idea of picking one problem at [a] time to look at solutions for it. Great job!
Very well run & moderated.
Smaller groups (have 4 instead of 2).
Maybe take a few minutes to discuss between groups at the end?
MORE TIME.
It was great!
Moderated very well! Format is effective!
More time!


What do you think FOR Boston should do next?

Pull together second groups to flesh out arguments.
More meetings (monthly?) if possible. Maybe with a new topic of discussion each time.
Create a post-doc email list for Boston-area postdocs: for career training opportunities, maybe for seminar announcements?, or maybe help create a Boston-area research seminar calendar? I realize that might be hugely ambitious…
Keep the ball rolling on this subject?
Have a specific action item list so that ideas actually produce change.
Gather data/collect info on postdoc career trajectory (e.g., through LinkedIn, surveys).
Organize summary sessions at individual universities to contact and inform all the people who couldn't make it, and people who didn't think about coming.
Follow-up event to generate actionable next steps and plan to continue to coalesce those who want to be engaged in getting to solutions.
Moderators at the main symposium should be more proactive in terms of keeping speakers on time and attendees with questions directed to [actual] questions not vague comments.
Continuing colding events, small and large, build collaborations nationally, connect with PDAs to hold more events.
Partner with NPA, AAMC GREAT group.
Breakfast seminars at universities - to get more individuals aware. Include faculty in discussions at universities.
Job fair.
It sounds like the NPA should be contacted to bring to their attention that most post-docs don't know the organization exists!
Make results from discussion groups available to postdocs - let us know what everyone said during the discussions.
Job fair!
Bring lay people into the discussion
How-to session - we've arrived at possible solutions - how do we put it into play.
Consolidate ideas into briefs for Congress, students, etc.
