# Supplementary material for: Shaping the Future of Research: a perspective from junior scientists
Source: F1000Res. 2015 Jan 9;3:291. Originally published 2014 Nov 28. [Version 2] doi: 10.12688/f1000research.5878.2 (PMC4304227; doi:10.12688/f1000research.5878.2)
Supplement: Update 1. Dataset of Future of Research Symposium — Legends describing each file can be found in the text file provided. Two new files have been added (Appendices 1A and 4), one file has been renamed (Appendix 1 has become Appendix 1B) and one file has been modified (Appendix 3). [file f1000research-3-6420-s0000.tgz › c07d938a-3bae-4814-adde-c49e101f85f8_Dataset_of_Future_of_Research_Symposium_update_1.zip!/Appendix 3B.odt]

APPENDIX 3B – WORKFORCE

Comment on Meaningful Solutions:
“Some, but is it realistic to think they will be supplemented?”

How could this session have been improved? 
“It was very well executed.  No need for improvement.”
“Venue was hard to hear everyone”
“No Suggestions. Well done.”
“Smaller room”
“Deeper discussion on fewer topics.”
“Make time for discussion.”
“We tended to deviate a lot toward the problems of postdoc training, which I don’t think was always very relevant to the problem of supply and deman.”
“The post-its/organization were a waste of time Could have been structured much more efficiently.  NEED to focus on a few, concrete actionable recommendations.”
“If at least one of the panel members were present the whole session.”
“Smaller Scope?”
“Drift of topics to several problems away from critical discussion points.”
“Smaller groups taking on narrower scope then coming back to larger group to present.”
“Smaller group.”
“More experienced/prepared moderators” (this person did say we addressed the objective really well)
“precise note-taking”
“increase physical distance between working groups-hard to hear our group over the other group.”
“Your effort shows and is appreciated.  Moderate confidently and feel free to politely quiet disruptive voices.”
“I thought the session was good.”
“Have more focused issues so that the discussions are more focused.”

 What should FOR do Next: 
“Have a follow-up on the issues brought up at this FOR with more concrete solutions already in place, and more resources available to become an informed citizen”

“Make contacts available and keep working groups going; continue social events.”
“Meeting with people who can actuallychange things: NIH/political/university officials.”
“Have these again and invite people in government and from the public.”
“Generally, raise awareness, specifically….?”
“Networking events (regularly)”
“Reach out to business/economic expertise is a source for a [additional] assumptions/new ideas.”
“Approach capital hill on improving the discrepancy between how cutting edge our R&D’s and the publics perception of how edge it is.  i.e. Tell the public that we will no longer be the front runners if don’t fund R&D.”
“Have a  ‘road show’; give talks at different Boston area institutions to educate postdocs about issues and options.”
“Maybe reach out to student and postdoc organizations (in general).”
