# Supplementary material for: Shaping the Future of Research: a perspective from junior scientists
Source: F1000Res. 2015 Jan 9;3:291. Originally published 2014 Nov 28. [Version 2] doi: 10.12688/f1000research.5878.2 (PMC4304227; doi:10.12688/f1000research.5878.2)
Supplement: Update 1. Dataset of Future of Research Symposium — Legends describing each file can be found in the text file provided. Two new files have been added (Appendices 1A and 4), one file has been renamed (Appendix 1 has become Appendix 1B) and one file has been modified (Appendix 3). [file f1000research-3-6420-s0000.tgz › c07d938a-3bae-4814-adde-c49e101f85f8_Dataset_of_Future_of_Research_Symposium_update_1.zip!/Appendix 3C.odt]

APPENDIX 3C – FUNDING

How could session have been improved?
Very well moderated
More open discussion, less post-it notes
Problem too big for time, start with more defined set of goals, eg. Set problems, with solutions, etc.
More time for discussion
More focused groups maybe
Maybe choosing one of the topics to more fully brainstorm (because of the short time)

What do you think FOR Boston should do next?
White paper, Quarterly meetings, focus meeting
Small discussion groups on key next steps
Future meetings with a single topic, discussion broke into different topics/ideas
If there is a follow-up meeting, focus on some of the open questions which arose.
Organize an event with congressmen/political players
Meet again! Meet with the higher ups and ask them our questions!
Boston wide postdoc union
