# Supplementary material for: Shaping the Future of Research: a perspective from junior scientists
Source: F1000Res. 2015 Jan 9;3:291. Originally published 2014 Nov 28. [Version 2] doi: 10.12688/f1000research.5878.2 (PMC4304227; doi:10.12688/f1000research.5878.2)
Supplement: Update 1. Dataset of Future of Research Symposium — Legends describing each file can be found in the text file provided. Two new files have been added (Appendices 1A and 4), one file has been renamed (Appendix 1 has become Appendix 1B) and one file has been modified (Appendix 3). [file f1000research-3-6420-s0000.tgz › c07d938a-3bae-4814-adde-c49e101f85f8_Dataset_of_Future_of_Research_Symposium_update_1.zip!/Appendix 3D.odt]

APPENDIX 3D – INCENTIVES

Were you able to arrive at meaningful solutions?  
Not really: The session was very interactive so we didn’t have time to complete the discussion.   But, great job!  
3:  Good ideas, but not sure how to make things actual incentives within the system.  
2:  Good ideas, not enough time.
2:  Interesting but meaningful/helpful?  Doable?

When making recommendations for change, how important is it for us to have each of the following:
Consensus:  3; Diversity of views 4:  You need to agree on some things, but I think it’s important that different perspectives are discussed.  
Consensus 2; Diversity of views 2:  Good/effective solutions, that’s what matters.

How could this session have been improved?  
Over-structured and prescriptive and too little time.
More time to discuss issues that were brought into consideration.  However I understand this was a broad screening session to initiate discussions.  Good job putting this together.
More clearly defined questions. 
I think it would have been helpful to focus on behaviors, and what incentivizes harmful behaviors in science to get to solutions/ideas more quickly.  Didn’t talk about stakeholders enough.  
I think there is an inherent challenge in trying to brainstorm problems and solutions in less than one hour and to come to consensus.  Maybe keep it as a brainstorming session, but less emphasis on thoroughly discussing issue??
More time on potential answers
There was some confusion over what questions were being asked – may be a timing issue b/c people came in late, but this made discussion difficult.  
Thanks guys!  Would help for us to bring the post-its with us so that the discussion could focus on prioritizing/evaluating solutions.  
Excellent workshop and symposium.  A bit more clarity on how to organize our thoughts and creating white paper would help next time.  
More time or more focused on a few issues – would help.  Or break groups into smaller # of people.
More interactive conversations/discussion than focusing on post its.  More verbal brainstorming than writing.  
More time or better time management.
Having more of these sessions.  Run it a little faster.  Better room setup so grouping takes less time.
Less time introducing (didn’t start really until ~1:20).
Longer time frames for activity or discussion.  
Post its were awesome -> different colors for “wants” and [incentives?].
Bring up this idea by a paragraph email to NIH and other responsible [institutions?].
Hard deadlines in brainstorming.  Less Bikeshedding [?].  Fewer categories.
Cut down # of opinions/tickets/labels and instead of categorizing, spend more time on solutions.
Too big a question for the time available.  Narrow the focus.  

What do you think FOR Boston should do next?  (If you have specific suggestions about people or organizations we should contact, please list.)
More discussions on gender and race equality in science.
Open letter to NIH – co-signed by PDA organizations and perhaps PIs/famous people.  Condense message.  
Hackathon.
More/longer workshops.  Have meeting w/people who could actually do things (NIH officials/policy makers, university heads).
Propel careers.  Grad student councils of different universities.  
Give talks @ each university to increase awareness among established PIs & promote discussion in broader community.
Talk to NIH/Congress.  
Meet again and discuss the issues that people seemed to have diverse views on.
Meet again; involve more people; bring business leaders.  
Publicize the blog as a place to discussion actions/incentives.
Maybe not immediately next, but FOR should eventually reach out to any other similar organizations that may (?) exist around the country.  Do they exist?
Make lists of search committee heads, speak w/them!
Expand to western MA.
