# Supplementary material for: Shaping the Future of Research: a perspective from junior scientists
Source: F1000Res. 2015 Jan 9;3:291. Originally published 2014 Nov 28. [Version 2] doi: 10.12688/f1000research.5878.2 (PMC4304227; doi:10.12688/f1000research.5878.2)
Supplement: Update 1. Dataset of Future of Research Symposium — Legends describing each file can be found in the text file provided. Two new files have been added (Appendices 1A and 4), one file has been renamed (Appendix 1 has become Appendix 1B) and one file has been modified (Appendix 3). [file f1000research-3-6420-s0000.tgz › c07d938a-3bae-4814-adde-c49e101f85f8_Dataset_of_Future_of_Research_Symposium_update_1.zip!/Appendix_1A.docx]

APPENDIX 1 – PRE-SYMPOSIUM SURVEY

In order to focus the aims of the workshops, participants were invited to complete an anonymous survey of their ideas about how science should be conducted and supported, and the problems they identified with the current system. In all, 409 people responded to the survey, although not all offered a response to all questions (raw data are available in Appendix 1). Respondents were primarily postdocs and graduate students, but also included administrators, faculty, industry, research assistants and undergraduates ( [Figure 1](" \l "f1)). The survey included five short-answer questions; while these responses are not amenable to quantitative analysis, we have summarized them below.

### Figure 1. Breakdown of the self-identification of respondents in the Future of Research Symposium registration survey.

### “What is the biggest problem facing the way science is conducted today?”

Answers focused on several key points, listed here by the frequency with which they were mentioned, starting with the most commonly cited problems.

- In the US, funding for basic science is inadequate to support long-term economic growth.
- The quality of the scientific results being produced is compromised by the current structure of research funding and execution.
- The research environment at present selects for proficiency at securing funding and publishing high-profile positive results, rather than rewarding scientific skepticism, curiosity, and balanced presentation of sometimes complex results.
- The current funding system is unnecessarily bureaucratic and insufficiently transparent, reflecting temporary political whims, and the duration of NIH grants is too short to support the lengthy explorations necessary to accomplish truly novel, beneficial basic research.
- The number of enthusiastic scientists competing for scarce funding encourages counter-productive levels of competition.
- Existing publication models exacerbate the problems arising from the inefficiencies of funding and the promotion of talent; journals disseminate research results in a periodic, page-limited manner that is outmoded in the internet era.
- There were many concerns about mentorship, trainee freedom and related issues indicative of an imbalance between the supply of qualified scientists and the demand for sufficiently-funded basic research positions.

### “What behaviors should be encouraged in scientists?”

The most common responses to this question (most commonly mentioned first) included:

- Collaboration, meaning interdisciplinary teamwork between scientists in different institutions and fields, as well as across boundaries of status and seniority.
- Openness in data, reagents, and evaluation of each other’s work.
- Integrity and ethical research practices, innovation, and risk-taking.
- Critical thinking in the reporting and reproducibility of results.
- Greater outreach to the public to improve non-scientists’ awareness of the most crucial results in recent research.
- Greater efficiency in the research process, as well as entrepreneurship, academic-industry partnerships, and more effective measurement of training and outcomes in basic research.

### “What does ideal scientific training look like?”

The overall consensus from responses to this question focused on the importance of teaching scientists how to solve problems with scientific methods in an ethical fashion.

- Training should be consistent across institutions, be multidisciplinary, and be independent of the race, sexual orientation, gender, gender identity or expression, national origin or cultural identification of its participants, to promote a community of diverse intellects.
- Mentorship should involve close interactions between mentor and mentee and should include well-defined expectations for both parties.
- A common request was for job security amongst researchers. Suggestions for implementation included a restriction upon the total number of PhDs awarded, an expectation of retirement based upon the age of PIs, an increase in the number of staff scientist positions supported by federal research funds, and more rigorous evaluation of scientists across institutions, from the undergraduate to the principal investigator level.

### “What should be the purpose of government funding for science?”

Respondents replied that government funding should balance an interest in both the long-term (basic) and short-term (applied) benefits of science.

- Industrial/commercial entities should assume responsibility for the advances that are most directly commercializable, while federal funding should address projects that are more prospective.
- Government funding should support public health and environmental health research that is otherwise not addressed by the immediate, private concerns of individual donors.
- To help support long-term research, some grants could be awarded to institutions, rather than individuals, to allow a community of researchers to decide among themselves which projects they find most meritorious.
- Within basic research, funding outcomes should be independent of expectations of immediate profitability. Many crucial advances within science have been made based on open-ended inquiry, driven by the curiosity of the individual personalities involved, and these contributions have subsequently proven essential to technical innovation.

Respondents also noted that excessive competition hinders collaboration and encourages non-productive duplication of experimental effort on select “hot topics”. The competition among qualified personnel for independent jobs is also highly inefficient in terms of wasted human capital.

### “The NIH has lost approximately 25% of its purchasing power over the last 10 years. Should the scientific workforce (i.e., make-up of the labor force: grad students, postdocs, senior scientists, etc.) adapt to this change, and if so, how?”

Only 13% of graduate students, 16% of postdocs, and 18% of faculty respondents suggested that the workforce should not adapt to the existing funding trends. Of those opposed to adaptation, established researchers (faculty) considered it more important to ignore fluctuations in funding. Most respondents suggested adaptation, using varying strategies. Several faculty respondents focused their attention upon lobbying congress and turning to public outreach in order to convince our fellow citizens of the importance of funding basic biomedical research. Other suggestions included:

- Reaching out to alternative funding sources, including state, local, and non-profit donors.
- Instituting longer timelines for approved grants.
- More direct funding by universities for employee researchers, encouraging smaller labs with more direct PI-trainee oversight.
- Greater understanding that non-academic careers are actually the major outcome for PhD holders, and support and encouragement for students and trainees who enter such careers.
- More transitional funds for entrepreneurial research and private-public partnerships.
- Changing the ratio of academic lab personnel between grad students, postdocs, technicians, and senior scientists.

The responses to this question were predominantly in favor of reducing the number of trainees per permanent position available in basic research, to steer funds towards more permanent positions, to seek alternatives to traditional funding sources (including private and nonprofit sectors), and to encourage greater regulation at the institutional and lab levels to address the efficiency of spending relative to the scientific research benefit produced.

Overall, the respondents’ concerns and criticisms centered on a few key themes; however, there was disagreement regarding which issues are most important to the future of groundbreaking and sustainable science. We considered these suggestions indicative of a general dissatisfaction with the current research paradigm, but not necessarily prescriptive of specific and comprehensive solutions. The output of this survey is informative in gauging the general opinion of educated, disciplined, and curious people pursuing science in the US. Practical adjustments to academic science were discussed in the workshops, described in the following sections.
