# Supplementary material for: Shaping the Future of Research: a perspective from junior scientists
Source: F1000Res. 2015 Jan 9;3:291. Originally published 2014 Nov 28. [Version 2] doi: 10.12688/f1000research.5878.2 (PMC4304227; doi:10.12688/f1000research.5878.2)
Supplement: Update 1. Dataset of Future of Research Symposium — Legends describing each file can be found in the text file provided. Two new files have been added (Appendices 1A and 4), one file has been renamed (Appendix 1 has become Appendix 1B) and one file has been modified (Appendix 3). [file f1000research-3-6420-s0000.tgz › c07d938a-3bae-4814-adde-c49e101f85f8_Dataset_of_Future_of_Research_Symposium_update_1.zip!/Appendix_3.docx]

APPENDIX 3 – EXIT SURVEY

At the end of each workshop, participants were asked to fill out a short exit survey (Figure 1). The survey was designed to address three objectives. First, in order to assess how well the workshop format was working and how it could be improved, participants were asked to rate on a five-point scale how well the session addressed the stated objective, and how well they were able to arrive at meaningful solutions.

The second objective of the exit survey was to determine whether or not participants felt they had reached a consensus during the workshop, and to gauge the importance participants placed on reaching consensus about these issues. As the primary official report arising from a symposium intended to give voice to early-career scientists, this document should reflect the views expressed at the conference. However, it was unclear whether that aim would be best accomplished by representing all or most of the opinions expressed during the workshops, or by presenting only those ideas about which participants had reached consensus. Participants were therefore asked to rate on a five-point scale whether or not their group reached a consensus and how important they thought it was to have either a consensus or a diversity of views regarding these issues.

Finally, we asked participants to list any specific suggestions they might have about next steps to be taken after the symposium, such as ideas about people or organizations we should contact. The results of the survey are summarized in Figure 2. The survey indicated that participants cared both about reaching a consensus and having a diversity of ideas. Participants also indicated that the workshops were more successful in generating multiple solutions than in finding unanimity.

To help us improve this session, please answer the following questions:

**How well did this session address the stated objective?**

Not at all	2	3	4	Very well

**Were you able to arrive at meaningful solutions?**

Not really	2	3	4	Yes, definitely

**Did your group reach a consensus, or did it include a diversity of views?**

Consensus	2	3	4	Diverse views

**When making recommendations for change, how important is it for us to have each of the following:**

**Consensus**	Not important	2	3	4	Very important

**Diversity of views**	Not important	2	3	4	Very important

**How could this session have been improved?**

**What do you think FOR Boston should do next? (If you have specific suggestions about people or organizations we should contact, please list.)**

**Figure 1. Text of the exit survey.**

**Figure 2. Workshop exit survey quantitative responses.**
