# Supplementary material for: Shaping the Future of Research: a perspective from junior scientists
Source: F1000Res. 2015 Jan 9;3:291. Originally published 2014 Nov 28. [Version 2] doi: 10.12688/f1000research.5878.2 (PMC4304227; doi:10.12688/f1000research.5878.2)
Supplement: Update 1. Dataset of Future of Research Symposium — Legends describing each file can be found in the text file provided. Two new files have been added (Appendices 1A and 4), one file has been renamed (Appendix 1 has become Appendix 1B) and one file has been modified (Appendix 3). [file f1000research-3-6420-s0000.tgz › c07d938a-3bae-4814-adde-c49e101f85f8_Dataset_of_Future_of_Research_Symposium_update_1.zip!/Appendix_4.docx]

APPENDIX 4 – MEDIA RESPONSE

The symposium received a wide variety of feedback and responses during and after the event, from both social media and the press, which continues to foster discussion.

During the symposium we engaged a large number of participants, both local and remote, using the [#FORsymp](https://twitter.com/search?f=realtime&q=#FORsymp&src=typd) hashtag on twitter, and the [@FORsymp](https://twitter.com/FORsymp) twitter account, to leave comments and ask questions. Figure 3 shows examples of significant tweets and questions received during the symposium. A collection of salient moments from the symposium was also gathered by Alberto Roca ( [@minoritypostdoc](https://twitter.com/minoritypostdoc)) into a [Storify](https://storify.com/MinorityPostdoc/forsymp?utm_content=storify-pingback&awesm=sfy.co_qibL&utm_campaign=&utm_medium=sfy.co-twitter&utm_source=t.co) ( Roca, 2014).

### Figure 3. Examples of significant tweets and questions from the symposium.

Comments were also received from interested non-local parties and the symposium was particularly well tracked in the UK and Australia, as illustrated in Figure 4. The comments reinforced that these problems are not unique to Boston or the US; this is part of the discussion we hoped to generate further afield.

### Figure 4. International interest over twitter.

Immediately after the symposium, significant attention was focused on the Future of Research in the publication of an article by Carolyn Johnson in the Boston Globe, “ [Glut of postdoc researchers stirs quiet crisis in science](http://www.bostonglobe.com/metro/2014/10/04/glut-postdoc-researchers-stirs-quiet-crisis-science/HWxyErx9RNIW17khv0MWTN/story.html?hootPostID=95f11e257c83450cbe021960c0ee3b84)” ( Johnson, 2014). The article remained the most viewed on the Boston Globe website for several days thereafter and generated a wide range of responses, including comments on the article itself:

- *“Not only is it unconscionable that these highly educated scientists - after being encouraged to pursue PhD’s by universities that know what career path difficulties lie ahead including years of being paid paltry annual salaries starting as low as $42,000, but it also makes being able to live a decent lifestyle and repaying their student debt impossible; now unforgivable even by bankruptcy”*.
- *“Boom-and-bust syndromes have plagued science and engineering markets for at least fifty years. Over and over, we’ve heard laments like those in the current article. Yet little, if anything, seems to have changed for the better. The problems, and the lack of genuine progress, have been vexing”*.
- *“One possible partial solution would be to treat academic research groups as long-term productive teams. Pay post-docs what they’re worth, and make their positions permanent mid-level research positions. There’s plenty of people in the pipeline who love doing science who don’t really want to be the head of their own lab, but we’re trained as though that’s the only option”*.

The Boston Globe itself compiled a [Storify](https://storify.com/BostonGlobe/research-community-responds-to) ( The Boston Globe, 2014) on responses to the article from social media, particularly through use of the hashtag [#lifeafterPhD](https://twitter.com/search?f=realtime&q=%23lifeafterPhD&src=typd), and published additional opinion pieces, including “ [Let’s change the system for postdocs](http://www.bostonglobe.com/opinion/2014/10/10/let-change-system-for-postdocs/3HWiKYcII1ZCSeBdm5XFwO/story.html)” and “ [Postdocs in limbo? Expand your options](http://www.bostonglobe.com/opinion/2014/10/10/postdocs-limbo-expand-your-options/vxdmaTBEszlWwwCIZAcUTP/story.html)” ( Fuentes-Medel, 2014; Kirshenbaum, 2014).

The article also generated further discussion on [reddit](http://www.reddit.com/r/GradSchool/comments/2ik9k2/glut_of_postdoc_researchers_stirs_a_quiet_crisis/):

- *“I think it is fairly absurd that the NIH estimate for number of postdocs working in the US has an error of ±15,500 people”*.
- *“Is it far-fetched to think that academia is headed toward a tipping point? Under the current situation, nobody currently in grad school or academia more broadly is going to recommend STEM as a career path to anyone, in particular their children”*.
- *“It’s frustrating because being a scientist is advertised as a stable job to people at every level of the education system until you’re actually in grad school”*.

There was also significant commentary on [Slashdot](http://news-beta.slashdot.org/story/14/10/07/2146258/glut-of-postdoc-researchers-stirs-quiet-crisis-in-science):

- *“If there was a genuine shortage [in STEM], you’d see sharp increases in salary levels. There’s just a shortage of qualified people willing to work for much less than they’re worth”*.
- *“Historically university posts were open to people with a BA (e.g. John Wesley and John Newman at Oxford in the 18th and 19th century). That it now takes a PhD and post doctoral work to get the same post means that we are training too many. Therefore the only solution is to row back on the PhDs being generated; given that governments are looking for money saving measures, this would seem an obvious starting point”*.
- *“It’s crazy that we have these vast hordes of people trained up and desperate to work hard for scientific progress. But our economy can’t find a way to provide them with jobs doing science”*.

Conversations on the LinkedIn group “PhD Careers Outside Academia”, “ [The Postdoc Holding Tank](https://www.linkedin.com/groupItem?view=&gid=1844342&type=member&item=5925347026446336002&trk=groups_items_see_more-0-b-ttl)” on October 5 ^th^ and “ [What we already know…](https://www.linkedin.com/groupItem?view=&gid=1844342&type=member&item=5925828140948533248&trk=groups_items_see_more-0-b-ttl)” on October 9th reiterated that this is not a new problem; the comments highlighted the dismal outlook among members of the community stemming from the lack of progress over the last decade. However, it is this very lack of change and the wider appreciation of the problem outside the academic community that we wish to target with the symposium and the publication of this document.

In the aftermath of the symposium, significant attention has been drawn to the event in academic media: in “ [Postdocs Speak Up](http://sciencecareers.sciencemag.org/career_magazine/previous_issues/articles/2014_11_05/caredit.a1400277)” ( Benderly, 2014), *Science Careers* magazine discussed the role of postdocs in advocating for themselves; an interview was featured in the scientific podcast [Beta Sandwich](http://betasandwich.com/podcast-archives/2014/11/2/064-life-after-your-phd-an-interview-with-future-of-research-co-founder-dr-kristin-krukenberg); and the National Postdoctoral Association published an article about the symposium in its publication, the [POSTDOCket](http://www.nationalpostdoc.org/images/stories/Documents/POSTDOCketArchives/POSTDOCket_Fall_14_v3.pdf).

We intend to continue bringing this conversation into the graduate student and postdoc community. Already, a [poster](http://f1000.com/posters/browse/summary/1097255) ( McDowell *et al.*, 2014b) has been presented at the [Out to Innovate 2014 Conference](http://www.noglstp.net/outtoinnovate/) held by the National Organization of Gay and Lesbian Scientists and Technical Professionals, Inc. ( [NOGLSTP](http://www.noglstp.org/)) and Out in Science, Technology, Engineering, and Mathematics, Inc. ( [oSTEM](http://www.ostem.org/)) at Georgia Tech (Atlanta, GA, November 8–9, 2014). The poster, which won the Leadership Poster Prize, generated a great deal of discussion regarding the intersection between postdoc and graduate student issues and issues facing underrepresented minorities.
