# Supplementary material for: Shaping the Future of Research: a perspective from junior scientists
Source: F1000Res. 2015 Jan 9;3:291. Originally published 2014 Nov 28. [Version 2] doi: 10.12688/f1000research.5878.2 (PMC4304227; doi:10.12688/f1000research.5878.2)
Supplement: Peer review history

# Peer review: Referee response for version 2

**DOI:** 10.5256/f1000research.6420.r7260

**Published:** 2015-01-12

**Authors:** Kenneth Gibbs Jr.

**Affiliations:** 1 National Cancer Institute, Washington, DC, USA

The report by McDowell *et al*. is much improved. I think it is appropriate for indexation. I hope the authors' found my comments helpful, and appreciate them taking many into consideration (I find the charts in particular quite helpful).

I have read this submission. I believe that I have an appropriate level of expertise to confirm that it is of an acceptable scientific standard.

# Peer review: Referee response for version 1

**DOI:** 10.5256/f1000research.6280.r6856

**Published:** 2014-12-11

**Authors:** Kenneth Gibbs Jr.

**Affiliations:** 1 National Cancer Institute, Washington, DC, USA

The report by McDonnell *et al.* aims to provide a summary of the Future of Research Postdoctoral Symposium that took place in October 2014. The work of this group is commendable, and has the potential to inform on-going policy debates about how to improve the research enterprise. The article has some interesting findings and recommendations. However, I feel there are two important issues that prevented me from accepting without reservation: focus and tone.

1. Focus. I found the manuscript, as written, very difficult to follow. My sense is that in an effort to be comprehensive, the focus became lost (as one who often writes long and unwieldy drafts, I have much sympathy). In my view, readability of the paper would be enhanced by organizing it in three sections:

- Context/Background. Here the authors can use data and other policy reports to describe some of the structural changes that have occurred in biomedicine in the past few years (e.g. stalled funding, growing number of trainees, etc.). They can then couple these changes with some of the issues that have resulted from this (e.g. an increased sense of competition to a level where it’s no longer helpful; the favoring of incremental science over exploration, etc.). From this point, they can say the purpose of this symposium: adding the voices of this groups of postdocs to the debate.
- Symposium. Clearly and concisely describe the symposium. List the four or five major goals/foci of the symposium (likely the workgroups). Then, for each, use a table to describe: 1. The central issue, 2. How postdocs describe the problem (only list the main points, and if possible include the percentages of postdocs conceptualizing the problem in that manner), and 3. The proposed solutions (with individual students, PIs/research groups, institutions, and funding agencies as their own column). Four charts would significantly enhance readability. All additional information can be put in the supplement.
- Summary recommendations. This could include a very brief nod to the media coverage (most of that should be in the supplement), the recommendations (currently the conclusions), and future recommendations.

2. Tone.  The paper read as a policy report, editorial, and meeting minutes all as one. There are many points where the authors make declarative statements but don’t offer any citations. Two examples of many include:

- “Specifically, the hyper-competition that * **we have all experienced** *, which stunts scientific curiosity and productivity, breeds fabrication and carelessness in the publication of data, and leads to a waste of valuable resources and intellectual capital, must be alleviated.”  It is not clear that all have experienced this and would make these conclusions.

- The paragraph starting with this sentence: “In spite of the number of years spent in pre- and postdoctoral training, * only a handful of scientists feel that they are adequately pre- pared for any job other than conducting research.*”  While career preparation is highly variable, it is an overstatement to say "only a handful feel prepared" for careers outside of research.  

The authors would be well served by making it clear when they are stating something that is a fact, versus when they are conveying the opinions of the participants. Additionally, sometimes the term “we” is used, and it’s unclear who it is referring to—the paper’s authors, FOR Symposium attendees, the broader research community. Please be clear to whom you are referring.

Finally, the manuscript has something that seemed to be a major contradiction.  In the executive summary, the authors say their report “represents a united voice of young biomedical scientists, conveying our concerns about the sustainability of the research enterprise and our hopes for change.”  However, on at least two occasions, they describe the challenge in reaching consensus among FOR Symposium participants

- “Overall, the respondents’ concerns and criticisms centered on a few key themes; however, there was disagreement regarding which issues are most important to the future of groundbreaking and sustainable science.”
- “Participants also indicated that the workshops were more successful in generating multiple solutions than in finding unanimity.”

Even if there is not a “uniform voice” the symposium findings are still important. Don’t feel the need to overstate conclusions.

Again, I feel the work is important. I fully expect the authors will be able to address my comments, at which point I would enthusiastically approve it.

I have read this submission. I believe that I have an appropriate level of expertise to confirm that it is of an acceptable scientific standard, however I have significant reservations, as outlined above.

# Peer review: Referee response for version 1

**DOI:** 10.5256/f1000research.6280.r6854

**Published:** 2014-12-09

**Authors:** Paula Stephan

**Affiliations:** 1 Georgia State University, Atlanta, GA, 30303, USA

The report provides an excellent portrayal of conditions faced by postdoctoral researchers and graduate students in the biomedical sciences in the United States and it makes a number of constructive suggestions for reforms that could make the system more “early career” friendly. For example, the authors advocate increased transparency so that doctoral students and postdoctoral scholars will know the career outcomes of those who proceeded them and a change in the ratio of individuals who staff labs between graduate students, postdocs, technicians and staff scientists. They also advocate a greater focus on facilitating the learning of soft skills while in training and a redirection of resources towards younger researchers and away from extremely senior researchers. (Over 7% of all NIH R01 supported PIs are currently 66 or older and about 3% are 36 or younger.) I would add to this list an increase in the salary of postdoctoral trainees: the current system, with its low pay for postdoctoral researchers, actively encourages an over reliance on postdoctoral trainees in the lab. I would also shift support of graduate students to training grants and away from graduate research assistantships. This would allow more control over the quality of training and the number of individuals trained. I would also note, as someone who has studied the funding of science and the biomedical work force for many years, that Vannevar Bush’s vision was to support graduate students and postdoctoral researchers on fellowships, not on graduate research assistantships. His vision was to train future researchers, not to provide support for trainees to staff labs during their years of training. It was only in the late 1950s and early 1960s that the system began to increasingly tilt towards supporting students as graduate research assistants. It is also interesting to remember that “over training” in the biomedical sciences has been a major issue for at least 40 years. As early as 1976, an NRC report concluded that a “slower rate of growth in the labor force in these fields [the biomedical sciences] was advisable.” And in 1998, the NRC report, “Trends in the Early Careers of Life Scientists,” chaired by Shirley Tilghman, called for restraint in the number of PhDs produced and increased use of training grants relative to graduate research assistantships.

I have read this submission. I believe that I have an appropriate level of expertise to confirm that it is of an acceptable scientific standard.
